# Manufacturing of Zinc Oxide Nanoparticle (ZnO NP)-Loaded Polyvinyl Alcohol (PVA) Nanostructured Mats Using *Ginger Extract* for Tissue Engineering Applications

**DOI:** 10.3390/nano12173040

**Published:** 2022-09-01

**Authors:** Hursima Izgis, Elif Ilhan, Cevriye Kalkandelen, Emrah Celen, Mehmet Mucahit Guncu, Hilal Turkoglu Sasmazel, Oguzhan Gunduz, Denisa Ficai, Anton Ficai, Gabriel Constantinescu

**Affiliations:** 1 Center for Nanotechnology & Biomaterials Application and Research (NBUAM), Marmara University, Istanbul 34722, Turkey; 2 Department of Bioengineering, Faculty of Engineering, Marmara University, Istanbul 34722, Turkey; 3 Department of Electronics and Automation, Vocational School of Technical Sciences, Istanbul University-Cerrahpasa, Istanbul 34500, Turkey; 4 Department of Metallurgical and Materials Engineering, Atilim University, Ankara 06830, Turkey; 5 Department of Medical Microbiology, School of Medicine, Marmara University, Istanbul 34722, Turkey; 6 Department of Metallurgical and Materials Engineering, Faculty of Technology, Marmara University, Istanbul 34722, Turkey; 7 Department of Inorganic Chemistry, Physical Chemistry and Electrochemistry Faculty of Applied Chemistry and Materials Science, University POLITEHNICA of Bucharest, Gh. Polizu 1-7, 011061 Bucharest, Romania; 8 National Center for Micro and Nanomaterials, UPB, Splaiul Independentei 313, 060042 Bucharest, Romania; 9 Science and Engineering of Oxide Materials and Nanomaterials, Faculty of Applied Chemistry and Materials Science, University POLITEHNICA of Bucharest, Gh. Polizu 1-7, 011061 Bucharest, Romania; 10 Academy of Romanian Scientists, Ilfov Street 3, 050045 Bucharest, Romania; 11 Department of Gastroenterology, Clinical Emergency Hospital of Bucharest, Carol Davila University of Medicine and Pharmacy, 050474 Bucharest, Romania

**Keywords:** ZnO NPs, electrospinning, antimicrobial effect, tissue engineering, wound dressing

## Abstract

In this research, as an alternative to chemical and physical methods, environmentally and cost-effective antimicrobial zinc oxide nanoparticles (ZnO NP) were produced by the green synthesis method. The current study focuses on the production of ZnO NP starting from adequate precursor and *Zingiber officinale* aqueous root extracts (ginger). The produced ZnO NP was loaded into electrospun nanofibers at different concentrations for various tissue engineering applications such as wound dressings. The produced ZnO NPs and ZnO NP-loaded nanofibers were examined by Scanning Electron Microscopy (SEM) for morphological assessments and Fourier-transform infrared spectrum (FT-IR) for chemical assessments. The disc diffusion method was used to test the antimicrobial activity of ZnO NP and ZnO NP-loaded nanofibers against three representatives strains, *Escherichia coli* (Gram-negative bacteria), *Staphylococcus aureus* (Gram-positive bacteria), and Candida albicans (fungi) microorganisms. The strength and stretching of the produced fibers were assessed using tensile tests. Since water absorption and weight loss behaviors are very important in tissue engineering applications, swelling and degradation analyses were applied to the produced nanofibers. Finally, the MTT test was applied to analyze biocompatibility. According to the findings, ZnO NP-loaded nanofibers were successfully synthesized using a green precipitation approach and can be employed in tissue engineering applications such as wound dressing.

## 1. Introduction

Tissue engineering is a rapidly expanding scientific field that uses cell and cell combinations with biomaterials and biologically active molecules to create, repair, and replace cells, tissues, and organs. It aids in producing materials that closely resemble the body’s native tissue/tissues [1,2]. Using micro and nanofabrication techniques to produce nanoparticles for tissue engineering has various advantages [3]. Nanotechnology can be utilized to create nanofibers, nanopatterns, and controlled-release nanoparticles for tissue engineering, which can replicate native tissues because the biomaterials to be designed at nanometer-size, such as extracellular fluids, bone marrow, and cardiac tissues [4]. Polymeric and composite nanofibers generated by electrospinning, which have exceptional features such as a high surface area/volume ratio and high porosity, are among the most widely used nanofibers in tissue engineering. The high versatility of this technique also allows the generation of inorganic nanofibres by using adequate precursors and conditions [5]. The electrospinning method is based on the electrostatic principle, which uses electrostatic attractive and repulsive forces in a high electric field to create nanofibers. The generation of fine fibers with high surface areas, ease of functionalization for varied purposes, superior mechanical qualities, and ease of processing are significant advantages of the electrospinning technique [6,7]. Because of these properties, nanofibers can be excellent materials for various biomedical and tissue engineering applications, including drug delivery, wound healing, artificial organs, and medical prosthetics [8]. Poly(vinyl alcohol) (PVA) is a water-soluble polymer that has been successfully used to create nanofibers by electrospinning. PVA is also a well-known noble metal particle stabilizer/capping agent, and thus, there is a high interest in developing PVA-based nanostructures [9]. PVA is a biopolymer that has excellent biocompatibility, biodegradability, fiber formability, chemical resistance, moisture absorbency, and swelling properties, making it perfect for use in wound healing [10,11]. Because they are non-toxic, biocompatible, and oxygen permeable, PVA nanofibers can absorb wound exudate and aid in tissue regeneration. In addition, PVA’s water-soluble properties make it an ideal polymer for adding nanoparticles [12]. In this study, ZnO NP nanoparticles loaded with ginger extract were obtained by the green synthesis method and were further embedded into biocompatible PVA nanofibers by the electrospinning method. Zinc oxide is a non-toxic and multifunctional inorganic oxide material with a vast range of applications in different fields [13,14]. The biological techniques for synthesizing ZnO NPs using various enzymes, microorganisms, plants, and their extracts have been suggested as effective, eco-friendly methods of nanoparticle synthesis [15]. This green synthesis, based on a bottom-up approach, is similar to chemical precipitation, where an extract of a natural product such as leaves of trees/crops or fruits is used to synthesize metal oxide nanoparticles loaded with the biologically active agents comprised in this extract [13]. These nanoparticles have an antimicrobial effect due to their high size/surface ratio. When the particles enter the cell, they interfere with cellular processes and adhere to the microbial surface, causing it to remain immobile [16]. ZnO is one of the most researched materials in tissue engineering because of its antibacterial properties and role in promoting cell growth, generation, and differentiation. These properties also allow it to be used in wound healing studies [17]. Using the disc diffusion method, the antimicrobial activity of ZnO NPs against various pathogens such as *Klebsiella*, *Streptococcus*, *Enterococcus*, and *E. coli* has been reported [18]. Dhandapani et al. demonstrated that ZnO NPs disrupted bacterial cell membrane integrity, reduced cell surface hydrophobicity, and decreased the transcription of genes resistant to oxidative stress in bacteria [15].

In this study, for the first time, ZnO NPs were synthesized by the green synthesis method using *Zingiber officinale* root extract and embedded into electrospun nanofibers at different concentrations for their potential in tissue engineering. Firstly, ZnO NPs synthesized by the green synthesis method were characterized by their antimicrobial activity and morphological properties. Then, ZnO NP embedded into PVA nanofibers at different concentrations were produced by electrospinning, and fiber diameters and morphologies were investigated by SEM. Chemical, mechanical, biocompatibility properties, swelling, and degradation behaviors were investigated to obtain a holistic overview of these nanostructures. The antimicrobial results obtained by disc diffusion showed that ZnO NP-loaded PVA nanofibers could be suitable for wound dressing studies with antimicrobial properties. As a result, ZnO NPs loaded with ginger extract and embedded into PVA nanofibers produced by environmentally friendly synthesis; electrospinning can be an alternative manufacturing method for the development of various nanostructures with applications in tissue engineering, especially for wound dressing in the treatment of chronic wounds.

## 2. Materials and Methods

### 2.1. Materials

Zinc acetate dihydrate (Zn(CH_3_COO)_2_·2H_2_O) was purchased from MERCK, Darmstadt, Germany. Polyvinyl alcohol (PVA, MW (89000–98000) with a deacetylation degree of 85–90%), Tween 80, sodium hydroxide (NaOH), phosphate-buffered saline solution (PBS; pH 7.4), and glutaraldehyde solution (12.5 wt% in H_2_O) were purchased from Sigma-Aldrich (St. Louis, MO, USA) and used without further purification. L929 *mus musculus* fibroblast cells were provided from Atilim University, Department of Metallurgical and Materials Engineering (Ankara, Turkey). DMEM-low glucose (Dulbecco’s Modified Eagle’s Medium-low glucose), FBS (fetal bovine serum), penicillin/streptomycin, L-glutamine, and phosphate-buffered saline tablets were bought from Amresco (Solon, OH, USA). 3-(4,5-dimethyl-2-thiazol)-2,5-diphenyl-2H- tetrazolium bromide (MTT) powder (99% Purity, *v*/*v*), Trypsin/EDTA solution at 0.25% (*w*/*v*), glutaraldehyde, dimethylsulfoxide (DMSO), hexamethyldisilane (HMDS) (99% purity, *v*/*v*), and trypan blue were obtained from Sigma-Aldrich (St. Louis, MO, USA).

### 2.2. Methods

#### 2.2.1. Preparation of the Zingiber Officinale Root Extract

Fresh ginger was taken from the market. It is thoroughly washed to remove contamination. The roots were air-dried by removing all moisture. The outer skin of the dried ginger was peeled and weighed 80 g. It was cut into small pieces and kept in an air oven at 50 °C for an hour. Completely dried roots were crushed in a mortar and pulverized. The 400 mL of deionized water was slowly added, and pounding continued, followed by filtering using no.1 Whatman paper and stored at +4 °C.

#### 2.2.2. Preparation of Zinc Oxide Nanoparticles

Briefly, 400 mL of 0.01 M zinc acetate dihydrate was prepared using deionized water. The prepared ginger extract was added to the zinc acetate dihydrate solution in different proportions (2, 2.5, 3, and 4%) slowly under magnetic stirring. To obtain pH = 12, 1.0 M sodium hydroxide was used. After reaching the desired pH, it was stirred for two hours until a white precipitate formed. This mixture was centrifuged at 10,000× *g* for 10 min. The supernatant portion was discarded, and the ZnO nanopowder was washed with deionized water and dried at 100 °C overnight. The resulting powder was collected and characterized appropriately.

#### 2.2.3. Production of the PVA/ZnO NPs Composite Gels

Briefly, 10 wt% PVA solution was prepared under a magnetic stirrer at 120 °C. After it was completely dissolved, the surface tension of the solution was decreased by adding 3% Tween 80 and mixed for 15 min at room temperature. The required amount of powdered ZnO nanoparticles loaded with ginger extract was used. After being dissolved in dH_2_O, the ZnO NPs were added to the solution and mixed on a stirrer at room temperature until a homogeneous solution was formed. All concentrations of the produced electrospinning solutions are shown in Table 1.

#### 2.2.4. Electrospinning Procedure of Nanofibers

The experimental setup consists of a needle, a syringe containing the polymer solution, a needle connected to the high-scale voltage generator, and an electrospinning (NS24) device. Solutions containing pure PVA and ZnO NP at 3 different concentrations (PVA/20%ZnO, PVA/40%ZnO, PVA/60%ZnO) were taken into the syringe as 10 mL. The flow rate was set at 0.6 mL/h, and the voltage was fixed at 25 kV. The working distance between the needle tip and the circular collector was 150 mm.

#### 2.2.5. Crosslinking Process of Nanofibers

All of the produced electrospun nanofibers were crosslinked by exposition to glutaraldehyde vapors. The nanofibers were positioned on top of the glutaraldehyde solution and incubated in an oven at 60° for 3 h [19].

#### 2.2.6. Fourier Transform Infrared Spectroscopy (FT-IR)

The chemical structure of the scaffolds was investigated by FT-IR (Jasco, FT/IR 4700, Pfungstadt, Germany). At room temperature, measurements were taken in transmission mode with a resolution of 4 cm^−1^ between 450 and 4000 cm^−1^. Thermo Nicolet 6700 FTIR spectrophotometer with Smart Orbit diamond ATR attachments was used to record the infrared (IR) spectra of our samples.

#### 2.2.7. Scanning Electron Microscope (SEM)

SEM was used to study the ZnO NP and nanofiber sizes and morphologies (SEM, EVO LS 10, ZEISS Istanbul, Turkey). The surface of prepared zinc oxide nanoparticles and fibers was coated with gold and palladium for 120 s with a sputter coating machine (Quorum SC7620, Laughton, UK). To obtain morphological properties and size differences, analyses of nanoparticles and fibers were conducted. After that, the average nanoparticle size and nanofiber diameters were analyzed using image software (Olympus AnalySIS, Tokyo, Japan).

#### 2.2.8. Mechanical Properties of Fibers

The tensile strength of fibers was determined and evaluated using a tensile test machine (SHIMADZU, EZ-LX, Kyoto, Japan). Before the tensile test, the thickness of the completely dry fibers was measured using a digital micrometer (Mitutoyo MTI Corp., Aurora, CO, USA). The upper and bottom parts of each sample were placed horizontally in the appropriate compartment of the instrument, and the standard deviation was calculated using standard % error bars.

#### 2.2.9. Swelling and Degradation Behaviours of Fibers

To determine swelling rates, the fibers were allowed to swell in phosphate-buffered saline solution (PBS; pH 7.4) at 37 °C. All fibers (PVA, PVA/20%ZnO, PVA/40%ZnO, PVA/60%ZnO) in 1 mL of PBS were kept on a 37 °C thermal shaker (BIOSAN TS-100, Riga, Letonya). The fibers’ initial weights (*W*0) were measured. For the swelling experiments, the samples’ wet weights (*Ww*) were weighed daily for up to 7 days. Equation (1) was used to obtain the swelling value (S) [20].

(1) $$S=\left[\frac{\left(Ww-W0\right)}{W0}\right]\times 100$$

The fibers were removed from the PBS medium and dried for 24 h at room temperature, weighing (*Wt*) daily for degradation testing. The deterioration index was calculated using Equation (2) [21].

(2) $$Di=\left[\frac{\left(W0-Wt\right)}{W0}\right]\times 100$$

#### 2.2.10. Antimicrobial Activity of ZnO NPs and Fibers 

Attenuation plantings of *S. aureus* ATCC 29213 and *E. coli* ATCC 25922 were made with 5% sheep blood agar (COS; Biomerieux, France) and McConkey agar (MCK; Biomerieux, France) media reduction. It was incubated at 35 °C overnight. Single colonies were falling on the medium in Mueller Hinton Broth (MHB; Biomerieux, France), 0.5 McFarland. Turbidity of 10–8 CFU/mL cell suspension was prepared. Suspended bacteria, Mueller Hinton E Agar (MHE; Biomerieux, France), was spread to cover the entire area of the medium for more than 10 min under UV light. Sterilized discs were evenly placed on the medium of each bacterial species. As a control antibiotic, ampicillin (2 g for *S. aureus* and *E. coli*) was used as a disc (10 g). The media were incubated for 16–20 h at ~35 °C. After incubation, the growth inhibition diameter around the disc was determined in mm. Medium planting was performed to reduce C. albicans ATCC 90028 Saburo dextrose agar (SDA; Biomerieux, France). It was incubated overnight at ~30 °C with colonies taken from the medium suspension at 0.5 MacFarland (10 6 CFU/mL) turbidity in saline. Fungal suspensions were spread on MHE agar. Each yeast has an equal number of discs placed at a distance. The control disc was a 70% ethanol-impregnated disc for both yeasts. It was incubated at ~35 °C for 20–24 h. In the incubation, the growth inhibition diameter was determined by measuring. Samples were placed on an antibiotic-free MHE agar medium for contamination assessment. After it was determined that there was no growth around the disc on the agar surface, samples were studied [22,23].

#### 2.2.11. Cell-Culture Studies

In vitro characterization of 20, 40, and 60% green-synthesized ZnO NPs, including pure PVA electrospun fibers, was performed via the L929 mouse fibroblast cell line. Firstly, the samples are UV-sterilized for 2 h. After UV sterilization, pure PVA, 20, 40, and 60% ZnO NPs, including PVA nanofibers, are placed into 96-well plates. The degree of attachment of the cells was examined by hemocytometer counting for the 3 h with 30 min intervals. The medium from each well was removed to eliminate unattached L929 cells. They were incubated for 15 min at 37 °C in Trypsin/EDTA solution (0.1% *w*/*v*). Finally, the remaining cells were counted using the trypan blue exclusion method for each time interval. The attachment results were given as the percentage of viable cells to initially seeded cells.

To determine the cell viability of the seeded fibroblast L929 on 20, 40, and 60% green-synthesized ZnO NPs, including pure PVA, an MTT assay was performed for 7 days. After incubation at 5% CO_2_, 37 °C, the growth media was removed from the specimens, and they were rinsed with PBS solution (pH 7.4) for one time. After rinsing with the PBS, 90 µL fresh medium and 10 µL MTT solution was added to each well, and then samples were incubated for 3 h. When the incubation was over, the MTT solution was discarded from each well, and 200 µL DMSO was added to dissolve the formed formazan crystals; then, the DMSO-added samples were incubated for an additional 1 h. Finally, absorbance values of the samples were measured via Dynamica LEDETECT96 microplate reader at 540 nm.

## 3. Results and Discussion

### 3.1. Characterization Process of Nanoparticles

**Figure 1:**
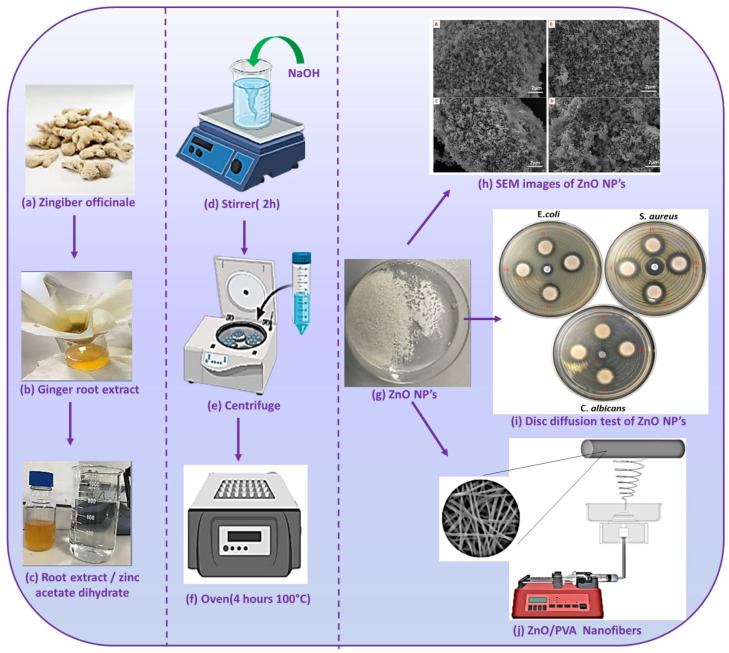
Production of ZnO NP by green synthesis method using ginger roots, SEM images of ZnO NP and antimicrobial result, and electrospinning of nanofibers by mixing zinc oxide nanoparticles with PVA polymer. (**a**) Cleaned and dried gingers; (**b**) filtered ginger extract, (**c**) mixing the ginger extract with zinc acetate dihydrate dissolved in water; (**d**) mixed in a mixer for 2 h, (**e**) centrifuge and discard the supernatant; (**f**) dried in the oven for 4 h; (**g**) the last version of ZnO NP; (**h**) SEM images of the obtained ZnO NP were taken; (**i**) the antimicrobial effect of the obtained ZnO NP was examined; (**j**) the obtained ZnO NP are mixed with PVA and nanofiber is obtained by electrospinning method.

As shown in Figure 1, ZnO NPs were produced and characterized by the green synthesis method using 2, 2.5, 3, and 4% ginger extract ratios. SEM images are shown in Figure 1h to understand the size, surface properties, and morphology of the produced nanoparticles. As a result of SEM, spiral-shaped nanoparticles of 100–130 nm were obtained. Figure 1i shows groups I, II, III, and IV representing 2, 2.5, 3, and 4%, respectively. The antimicrobial properties of nanoparticles obtained by using ginger extract at different rates were examined by the disk diffusion method, and the results are given in Table 2. Clear inhibitory zones developed in *S. aureus, E. coli*, and *C. candida* species. Since 2% ZnO NPs formed a higher inhibition zone, this ratio was continued in the later parts of the studies. PVA/ZnO nanofibers were created using the electrospinning technique with the addition of 2% ZnO NPs concentration and creating a composite structure with PVA polymer in three different ratios (20, 40, and 60%).

### 3.2. Fourier Transform-Infrared Spectroscopy (FT-IR)

**Figure 2:**
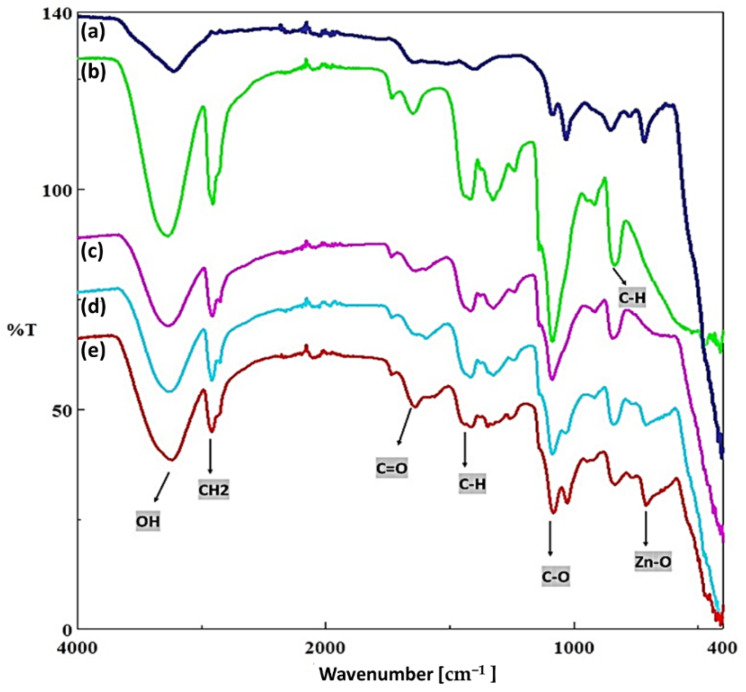
FT-IR spectrum of pure PVA, ZnO NPs, and PVA/ZnO composite nanofibers. (**a**) ZnO NP, (**b**) PVA fibers, (**c**) PVA/20%ZnO NPs solution, (**d**) PVA/40%ZnO NPs solution, and (**e**) PVA/60%ZnO NPs solution.

Fourier Transform Infrared (FTIR) spectroscopy was also used to evaluate the chemical interactions between the PVA and ZnO NPs (Figure 2). PVA have the main characteristic peaks at ~3268.75 cm^−1^ (OH group, NH amino group), ~2910 cm^−1^ (CH stretch vibration), ~1646.91 cm^−1^, ~1417.42 cm^−1^ (CO), ~1326.79 cm^−1^ (CH bending), ~1261.22 cm^−1^ (C=O vibration), ~1085.73 cm^−1^ (CO group), ~917.95 cm^−1^ (CC stretching), and ~ 821.53 cm^−1^ (CO stretching) [24]. The weak and broad bands seen around 3500 cm^−1^ in all groups indicate associated -OH groups. The presence of zinc oxide nanoparticles was confirmed by the FTIR spectrum of PVA/ZnO composite nanofibers, which showed a band around 600 cm^−1^ in addition to other peaks related to PVA due to Zn-O stretching [25]. C-H and C=O groups have weak peaks at 1500 cm^−1^ and 1700 cm^−1^, respectively, while –CH_2_ and C–O groups have sharp peaks at 1100 cm^−1^ and 2700 cm^−1^. When comparing the peaks formed by similar groups of ZnO and PVA, the PVA peaks were sharper and more distinct, whereas the Zn–O peaks were larger and smaller. The PVA/ZnO groups formed peaks in the mean of the other two groups at various concentrations [26,27]. Kandulna and Choudhary showed that O–H bonds formed between PVA and ZnO in their studies. The H bonds that are created between the H groups in the PVA matrix’s open chemical structure and the O atoms in the structure of the ZnO NPs are what allow for contact, according to the analysis of the structure. These interactions can also be caused by H bond interactions between –OH groups, as there are –OH groups in both groups, as seen in FTIR in Figure 2, because the H bond can interact very quickly, so it can be quickly broken and reconnected between different groups [28].

### 3.3. Scanning Electron Microscope (SEM)

**Figure 3:**
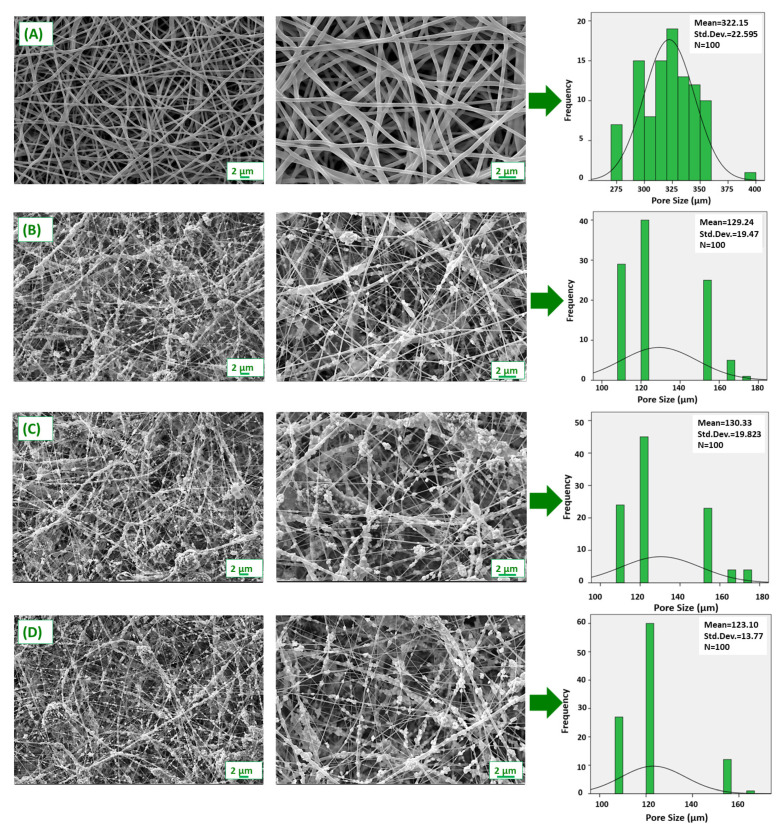
SEM images and pore size histogram of (**A**) pure PVA, (**B**) PVA/20%ZnO, (**C**) PVA/40%ZnO, and (**D**) PVA/60%ZnO nanofibers.

The surface properties and nanofiber diameters of PVA/ZnO fibers were investigated using SEM (Figure 3). According to the images, the pure PVA nanofiber patch exhibited homogeneous, continuous, and bead-free morphologies compared to the ZnO NP-loaded patches. These smooth and homogeneous morphologies formed a porous network that allowed nutrients and oxygen to diffuse to the linked cells [29]. As seen in Figure 3, the diameters of all fibers were measured, and histogram graphs were created. While the average fiber diameter of the pure PVA fiber was 322.15 nm, significant changes were observed in both the diameters and surface morphologies of the fibers after the addition of NP. The mean fiber diameters of PVA/20%ZnO, PVA/40%ZnO and PVA/60%ZnO fibers were calculated as 129.24, 130.33, and 123.10 nm, respectively. While a significant reduction in the diameters of the fibers was observed, the presence of NPs was observed in the fiber morphologies. It can be attributed that ZnO NPs added in high concentrations increased the solution viscosity compared to the pure PVA solution, which significantly reduced the diameters of the nanofibers. This occurs when metal salts transform into metal oxides and fibers contract [30]. NPs embedded in nanofibers also show that nanofiber diameter and nanoparticle size are appropriate. Because of its antimicrobial activity, the dense distribution of ZnO NPs in the fibers is critical for the wound’s functionality [31]. Due to the tendency of nanoparticles to reduce excess surface energy by forming large particles, some ZnO agglomerations can be seen in some areas, which is also due to the high polarity of water, as shown in Figure 3 [32]. Comparing the distribution of the ZnO NPs with the size of the pathogen strains, it is obvious that these pathogens cannot adhere to the patches without being in contact with these nanoparticles, and thus, it is expected that bacterial adherence will not be possible, and these patches can act for as an anti-adherent, antibiofilm dressing, and because the pores generated by the disposal of the fibers are again small, these patches can act as barriers against the microbial strains. 

### 3.4. Mechanical Properties of Fibrous Patches

**Figure 4:**
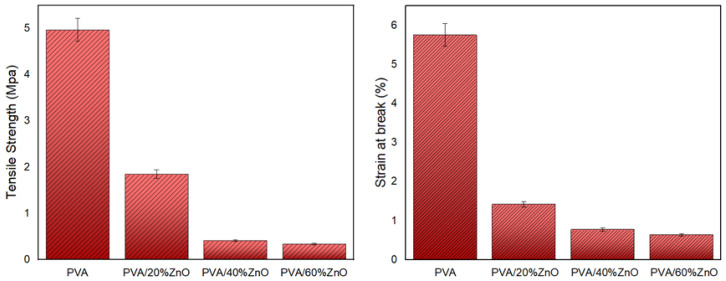
Mechanical parameters of all fibers: the tensile strength (Mpa) and strain break (%).

The mechanical properties of the produced patches were analyzed by performing the tensile test (Figure 4). While the mechanical strength of pure PVA nanofiber reached around 4.5 MPa, a decrease was observed in its mechanical strength as ZnO NPs were added to it. Similar behavior is observed in elongation at break. Inorganic ZnO NPs have the potential to break the bonds between PVA chains, lowering the durability of the material, but can also be associated with the thinner (strangled) fibers induced by the presence of these nanoparticles. All of the PVA/ZnO groups in this study had a positive tensile strength. Because of the covalent bonds, the tensile strength is high, but as the amount of ZnO in the mixture increases, the intermolecular hydrogen bonds become stressed, causing hardening and ruptures. Because the interface interactions of ZnO NPs are weak, they cannot be said to have a positive effect on the strength of the PVA matrix [33].

### 3.5. Swelling and Degradation Behaviours of Fibers

**Figure 5:**
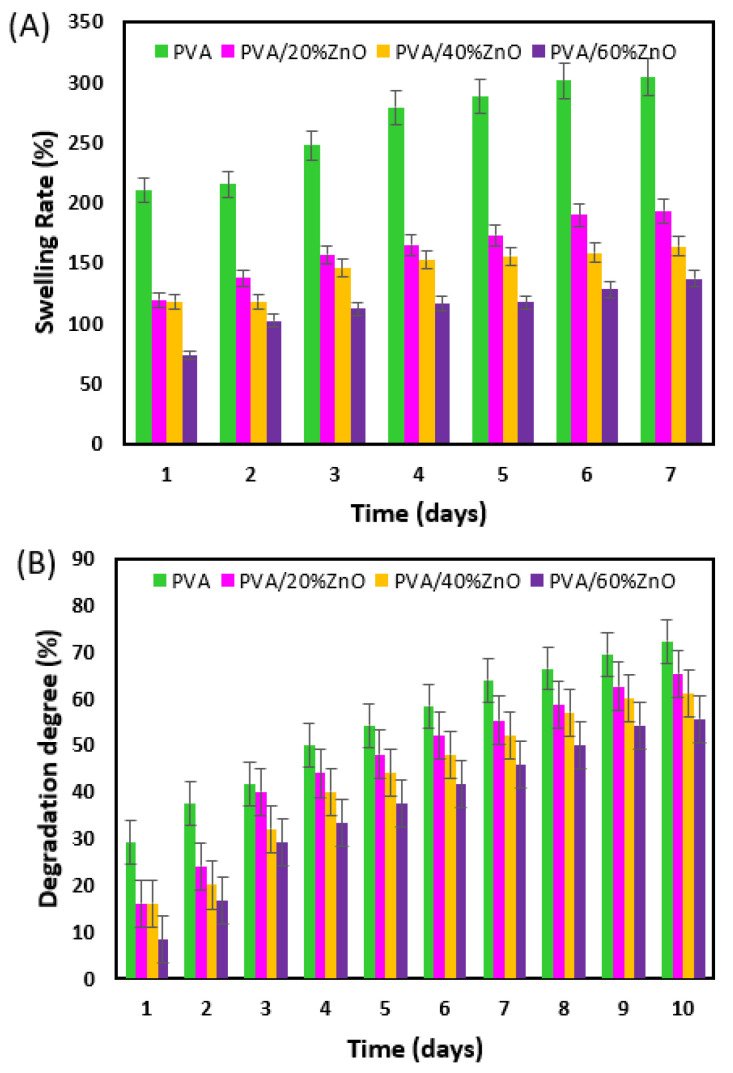
Swelling (**A**) and Degradation (**B**) kinetics of nanofibers in PBS 7.4 at 37 °C.

Wound dressings in tissue engineering must have a high water absorption and moisture capacity, which is critical for wound infection prevention and healing [34]. The ability of scaffolds used in tissue engineering to absorb water aids in delivering nutrients and oxygen to the interior [35]. The dressing’s swelling capacity is a practical and crucial metric for wound infection control [36], and it correlates the biological features of the wound with the dressing’s physicochemical qualities. Different simultaneous impacts can be used to understand the dynamics of swelling. The contours of the penetration uptake vs. time curve diverge from the classical Fickian model more frequently. When this happens, the sorption process involves synchronous mesh segment relaxation due to the advancing solvent front, resulting in material plasticization and a considerable increase in volume [37]. The ionization of negatively charged groups also affects the swelling of nanofibers. Figure 5A demonstrates that both PVA and PVA/ZnO fibers at different concentrations showed greater swelling ability from day 1 to day 7. Although the pace of swelling reduces as ZnO NP is added, it maintains the needed degree of swelling for wound healing [38]. As the amount of ZnO NP in the fiber increased, it was observed that the swelling ratio of the nanofibers decreased. This can be attributed to the inhibition of water penetration by ZnO NP occupying some voids in the hydrogel network, restricting its expansion [39]. Marvizadeh et al. [40] also discovered that adding ZnO NPs to the starch/gelatin nanocomposite film improved its hydrophobicity.

A crucial criterion for tissue engineering is the degradation of structures exposed to PBS and the loss of bulk over time [41]. For ten days, the degradation of PVA/ZnO fibers at four different concentrations was measured every 24 h. Due to the maximal swelling capacity, the PVA nanofiber displayed stronger degradation behavior than other nanofibers after 10 days, as shown in Figure 5B. The nanofiber’s capacity to swell allows the amorphous chains to move more freely, enabling the fibers to break away from the framework and accelerating weight loss [42]. According to the data, the degradation process was proportional to swelling in all four scaffolds.

### 3.6. Antimicrobial Activity of ZnO NPs and Fibers 

ZnO NPs were synthesized as powders using ginger extract at levels of 2, 2.5, 3, and 4%. *E. coli* (ATCC 25922 (mm)), *S. aureus* (ATCC 29213 (mm)), and *C. albicans* (ATCC 90028 (mm)) were used in Figure 1h to test the antibacterial activity of the synthesized ZnO NPs. There is a zone of inhibition diagram available. The inhibitory zones created are measured and displayed as a table in Table 2. Because all ginger ratios employed produce an inhibitory zone with the same length, ZnO NPs with 2% ginger was chosen, a composite structure was made using PVA polymer, and nanofibers were created using the electrospinning technique. Results showed the same magnitudes when saturation was attained following a particular ginger extract [31].

**Figure 6:**
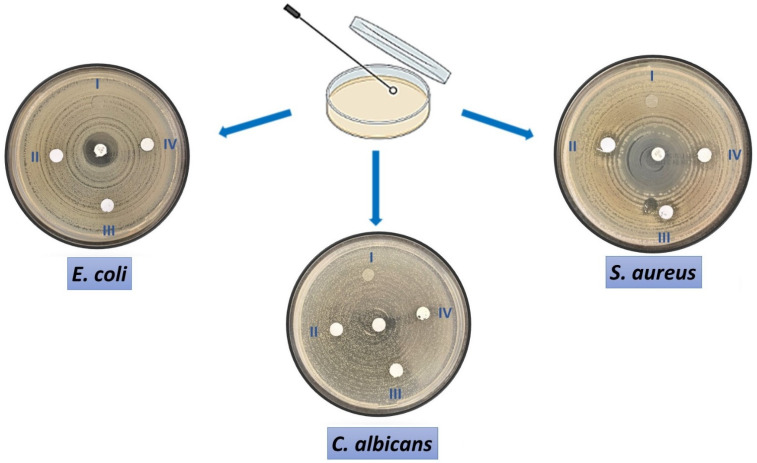
Antimicrobial activity of four different concentrations of PVA/ZnO fibers in *E. coli*, *S. aureus*, and *C. albicans* by disk diffusion method.

Figure 6 shows the antimicrobial activity of both control and ZnO NP-loaded nanofiber patches against the reference microorganisms, *E. Coli* (ATCC 25922 (mm)), *S. Aureus* (ATCC 29213 (mm)), and *C. Albicans* (ATCC 90028 (mm)). Antimicrobial activity is determined by measuring the diameter of microorganisms inhibition around the fiber discs, as shown in Figure 6. The number of particles per surface area/volume will be low because the nanoparticles will be dispersed over a large area in the polymer solution. The nanoparticle ratios were kept high to see antimicrobial activity. No zone formation is observed in terms of *E. coli* microorganisms in pure PVA, PVA/20%ZnO, and PVA/40%ZnO fibers. However, a clear zone with a diameter of 7 mm was observed in the PVA/60%ZnO nanofiber, but, for *S. aureus*, a 12 mm inhibition zone was observed in the other nanofibers, except for pure PVA. It can be said that the produced patches loaded with nanoparticles show the necessary antimicrobial effect for the antimicrobial *S. aureus*. At the same time, for *E. coli*, the inhibition is observed with the increase in the amount of NP. The difference between Gram-positive and Gram-negative bacteria cell wall architectures accounts for the stronger antibacterial action against *S. aureus* than *E. coli.* On the other hand, Gram-positive bacteria have a strong cell wall structure with numerous layers of peptidoglycans, and Gram-negative bacteria have a more complicated cell wall structure, making them more resistant to antibacterial agents [43]. One of the greatest methods for creating dressings with antibacterial characteristics is the insertion of metal nanoparticles and metal oxide into the polymeric membrane structure. Hydrogels, nanocomposites, and nanofibers are examples of materials with high porosity, great gas permeability, and high surface-to-volume ratios. These are necessary for wound healing because they promote better skin regeneration, hemostasis, exudate clearance, and hydration [44]. Wounds are exposed to bacteria that make healing difficult and induce inflammation. The use of antibacterial agents accompanying biocompatible and biodegradable polymers to prevent infection has been demonstrated in studies by Homaeigohar and Boccaccini. The Zn group in ZnO NPs accelerates the wound healing process by acting on enzymes that provide cell division and proliferation. In addition to the antimicrobial activity of ZnO NPs, other properties that affect the wound healing process are fibroblast proliferation, induction of angiogenesis, and cell migration [45]. A greater ZnO concentration results in more reactive oxygen species (ROS), such as superoxide anion (O_2_^−^), hydroxide (OH^−^), and hydrogen peroxide (H_2_O_2_), which are related to the effect of ZnO NPs concentration on antibacterial effectiveness. The presence of O_2_ and OH, which are too big to enter the internal matrix and instead remain on the bacteria’s outer surface, compromises the integrity of their cell walls. On the other hand, H_2_O_2_ molecules can penetrate the bacterial cell wall, build up, and cause a high level of oxidative stress inside the cell, which results in cell death. The thicker peptidoglycan layer of Gram-positive bacteria such as *S. aureus* causes more charge transitions created by ZnO NPs, which disrupts the cell wall. Reactive oxygen species can attach to the peptidoglycan layer less readily in Gram-negative bacteria such as *E. coli* than in Gram-positive bacteria due to the latter’s thicker coating. The bacterial cell wall structure is the reason why the inhibitory zone created by *E. coli* is less than that of S. *aureus*, as seen in Table 3 [46]. In addition, ZnO NP has been reported to have a high fungicidal effect on *C. albicans* [47]. In this study, no inhibition zone formation was observed in *C. albicans* when ZnO NP was embedded in the PVA patches. However, it is expected that ZnO NPs inhibit the growth of *C. albicans* in a concentration-dependent manner and prevent *C. albicans* growth with the increase in the amount of ZnO [47]. 

### 3.7. Cell-Culture Studies

#### 3.7.1. Cell Attachment

**Figure 7:**
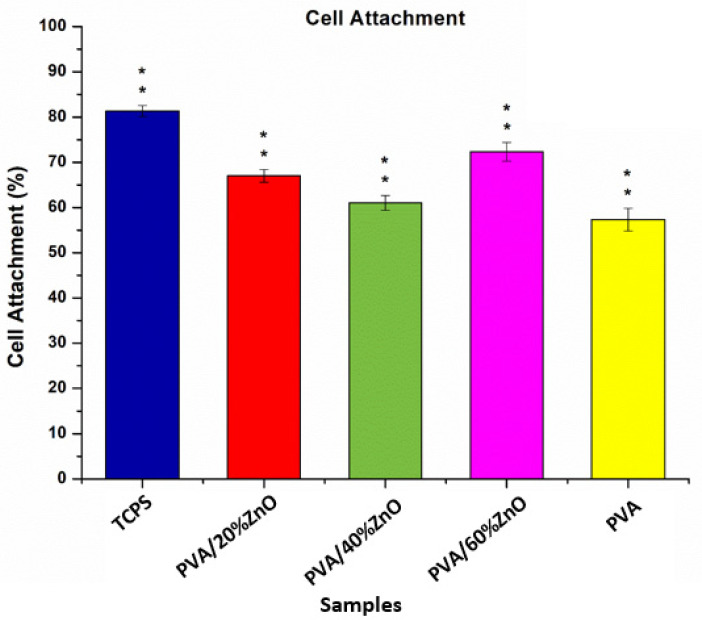
Percentage of cell attachment on the TCPS as control and electrospun fibers. Statistical significance ‘*p*’ is shown as *p* < 0.05 = ‘*’ and *p* < 0.01 = ‘**’.

Figure 7 shows the percentage of viable cells per seeded cell. Cells successfully attached to Tissue Culture Polystyrene (TCPS), pure PVA fibers, PVA/20%ZnO and PVA/40%ZnO PVA/60%ZnO electrospun fibers. Figure 7 indicates that TCPS showed the highest cellular adhesion with 81%. Although PVA/60%ZnO has less cell viability than PVA/20%ZnO, it has more cellular adhesion. Since pure PVA samples have smoother surfaces and larger diameters, all ZnO-added PVA samples showed better cellular adhesion. Adding ZnO increased the surface roughness and decreased the diameter, which can also be associated with the release of Zn^2+^, which is well known to be also beneficial for cell division [40].

#### 3.7.2. Cell Viability

**Figure 8:**
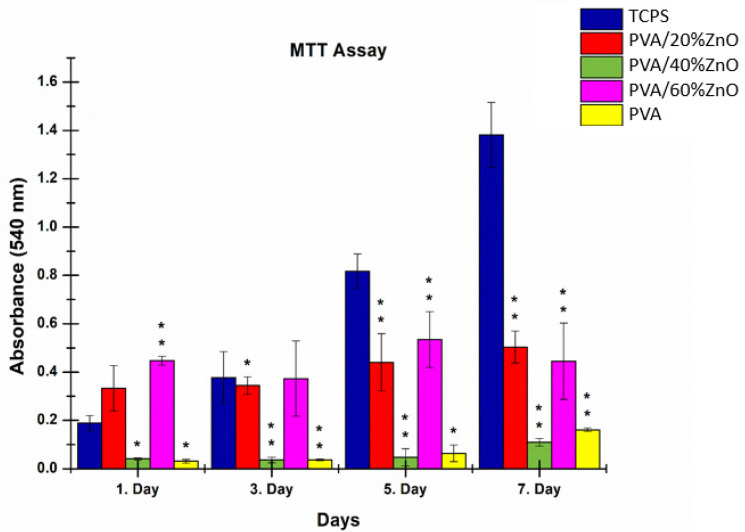
Viability of L929 cells cultured on pure PVA and 20, 40, and 60% ZnO included PVA electrospun fibers. Statistical significance ‘*p*’ is shown as *p* < 0.05 = ‘*’, *p* < 0.01 = ‘**’.

The viability of the L929 cells on the PVA-based patches loaded with 20, 40, and 60% green-synthesized ZnO NPs, as well as on pure PVA electrospun patches, were determined. The results of the MTT assay are presented in Figure 8. The developed pure PVA fibers, PVA/20%ZnO and PVA/40%ZnO electrospun fibers, did not show any cytotoxic effect on the L929 cells for 7 days. PVA/60%ZnO fibers were not cytotoxic to L929 on 1st day. However, on the third day, the cell viability the cells decreased for PVA/60%ZnO. Although the absorbance value of the PVA/60%ZnO is more than the PVA/20%ZnO for the first day, the absorbance of the PVA/20%ZnO increased with each day, and on the seventh day, the cell viability of PVA/20%ZnO is higher than both PVA/40%ZnO and PVA/60%ZnO. The PVA/40%ZnO sample has more fiber diameter, thus less porosity than other samples, and higher porosity increases the cell adhesion to the surface [48,49]. Even though the cell viability for the PVA/40%ZnO increases each day, it has less cell attachment than PVA/20%ZnO and PVA/60%ZnO; thus, the overall absorbance value is in between these samples. Patrón-Romero et al. and Resmi et al. reported that the higher concentrations of green-fabricated ZnO NPs decrease the cell viability of L929 cells [50,51]. According to the given knowledge, PVA/20%ZnO containing electrospun fibers are preferable for the L929 cells, and it can be assumed that PVA/60%ZnO increases cell attachment more than other samples. Still, the total number of cells fluctuates due to the more leakage of ZnO NPs as well as Zn^2+^ release into the environment. 

## 4. Conclusions

In this study, zinc oxide nanoparticles were synthesized using the root of the zingiber officinale plant extract by a green reduction synthesis route and were further successfully loaded into PVA nanostructured mats. The fiber diameters of ZnO NP-loaded nanofibers decreased from 322 nm to 130 nm, which is ideal for tissue engineering. Adding ZnO NPs increased the cellular adhesion in cellular attachment studies and proliferation of L929 fibroblast cells after 7 days of incubation in the MTT assay. The antimicrobial activity is good against the standard strains. As a result of the analysis, it is expected that the produced antimicrobial ZnO NP-loaded electrospun nanofibers will be used as an alternative in various tissue engineering applications. Further works will be devoted to the evaluation of the synergies, which can be obtained by optimizing the nature of the natural extract, which is used to obtain ZnO nanopowder. 

## Figures and Tables

**Table 1:** Percentages of ZnO NPs and PVA of solutions prepared at different concentrations.

	ZnO NPs Content (wt%)	PVA Content (wt%)
PVA	0	10
PVA/20%ZnO	20	10
PVA/40%ZnO	40	10
PVA/60%ZnO	60	10

**Table 2:** As a result of the disc diffusion (antimicrobial test) test, the inhibition zones created by the zinc oxide nanoparticles were measured in mm and listed in the table. These inhibition zones were created by adding ginger extract to the prepared zinc oxide solution at various rates (2, 2.5, 3, and 4%).

Sample Number	Sample Name	*S. aureus* ATCC 29213 (Diameter-mm)	*E. coli* ATCC 25922 (Diameter-mm)	*C. albicans* ATCC 5314 (Diameter-mm)
I	2%	22 mm	20 mm	14 mm
II	2.5%	22 mm	19 mm	14 mm
III	3%	22 mm	19 mm	14 mm
IV	4%	22 mm	19 mm	14 mm
V	%70 Ethanol	-	-	15 mm
AMP 2	Ampicillin 2 µg	18 mm	-	-
AMP 10	Ampicillin 10 µg	-	12 mm	-

**Table 3:** Measurement of inhibition zones formed due to the antimicrobial activity of ZnO NPs and 4 different concentrations of PVA/ZnO fibers in *E. coli*, *S. aureus*, and *C. albicans* by disk diffusion method.

	*E.coli* ATCC 25922 (mm)	*S. aureus* ATCC 29213 (mm)	*C. albicans* ATCC 90028 (mm)
ZnO NPs	22 mm	20 mm	15 mm
I-PVA	0	0	0
II-PVA/20%ZnO	0	12 mm	0
III-PVA/40%ZnO	0	12 mm	0
IV-PVA/60%ZnO	7 mm	12 mm	0
(AM)-Ampicillin	12 mm (10 µg)	30 mm (2 µg)	14 mm (%70 ethanol)
